# *Back to School after Corona Virus Disease of 2019*: New Relationships, Distance Schooling, and Experienced Routine

**DOI:** 10.5334/cie.71

**Published:** 2023-09-15

**Authors:** Maria Gabriella Pediconi, Michela Brunori, Savino Romani

**Affiliations:** 1 Department of Economics Society Politics (DESP), University of Urbino, Urbino, IT

**Keywords:** affective relationships at school, coronavirus pandemic, distance schooling, pre-adolescents and adolescents, schooling routine

## Abstract

The Corona Virus Disease of 2019 (COVID-19) pandemic has upset the students’ daily routine, forcing them at first into a sudden transition to distance learning and then to a return to school modelled on the basis of infection containment measures. The present research involved 157 students from schools in central Italy with a mean age of 13.58 years old to investigate the affective impact of the pandemic on the school experience and its components (recess, oral testing, relationships with classmates, and relationship pupils-teachers). The results show that only a few have experienced school interruption in a traumatic way: they have appreciated neither distance learning, nor the return to school; for these teenagers, the school of the past has died. Other adolescents and pre-adolescents tried to replace the face-to-face mode with distance learning, maintaining certain attention to the school even during the quarantine. However, the online mode did not keep its promise. Those who have invested more in digital innovation find it difficult to return to normality today. For all of them, socialization mediated by school experience is decisive in supporting the return to ordinary life after the pandemic.

## COVID-19 at School

Preadolescents are faced with major and rapid physical transformations that engage them in constant vigilance concerned with bodily characteristics (Petter 1990; Senín-Calderón et al. 2017). Parallel to the changes in the timbre of the voice, the appearance of secondary sexual characteristics and the menstrual cycle in girls, the remarkable growth of stature especially in boys, there is a metamorphosis at a psychological, cognitive and affective level that initiates the appearance of anxieties and insecurities related to the sense of estrangement and acceptance of the new self-image (Bonanno, n.d.). Moreover, for preadolescents, parenting figures are no longer the only affective referent, social comparison processes emerge and childhood identifications begin to integrate with the significant objects of the present. In this particularly turbulent emotional dimension (Steinberg 2005) concerns with interpersonal identity become acute, increasing preadolescents’ vulnerability to interpersonal influences. However, if in puberty strong residues of childhood dependence remain, in adolescence the detachment from parental figures is decidedly more marked, the adult community is perceived as “other than oneself” while the peer group becomes the counterculture opposed to the adult world in which the adolescents recognize themselves (Lutte, 1980). At the heart of the adolescent’s evolutionary tasks is acquiring a sense of individuality and consolidating an autonomous identity, and the surrounding social environment plays a fundamental role in this (Erikson, 1995).

### Routine and Affects at School

School is where pre-adolescents and adolescents spend most of their time, and it provides an ideal setting to acquire social and emotional skills, as well as behaviours that translate into positive real-life health outcomes. In addition to providing knowledge and learning, schools offer an appropriate environment to promote healthy functioning and well-being for the youth, allowing them to build their identity in a constant testing both in educational and affective terms.

Alerby (2003) elucidated pupils’ experience of school and interpreted the meaning of their experiences showing how learning and knowledge, social relations, orderliness and rules, subjects, feelings and time-dimensions are the main aspects of youth’s school experience. In particular, he demonstrated that school is constituted by

*multifaceted experiences which have many nuances, such as a lack of time to accomplish schoolwork, the importance of breaks and relations between friends, the importance of the relations between the teacher and the pupils, the fact that rules are decided upon over the pupils’ heads and the fact that learning things and developing knowledge are positive even if school is sometimes experienced as boring*. (p. 17)

The social structure of school consists of the patterns of social relationships occurring between the members of the school. On the one hand, we find the pupils and teachers relationship, through which adolescents can integrate their concept of the reference figure (Cooper, 2002; Guo et al., 2020; King, 2017; Uitto & Syrjälä, 2008; Wentzel, 2009). Socio-relational competencies are an essential component of teachers’ professionalism linked to the well-being of their students: positive relationships between teachers and pupils have a positive association with students’ self-esteem and future expectations (Marini et al., 2023). On the other hand, we find the peer-to-peer relationship, indispensable for the construction of one’s own identity (Ferrer & Fugate, 2003). The school is a promoter of new acquaintances and friendships, in particular recess is a really interesting space in the school day because it’s a time when there could be a lot of academic, social and emotional physical growth happening. It is characterized as that moment in which students have the opportunity to engage with their peers, get to know each other, play together, tell each other about school, extracurricular experiences and create new bonds (Alerby, 2003), which in many cases accompany the adolescent also in the subsequent stages of life.

The COVID-19 pandemic has forced the school system to a sudden and unprepared transition to distance learning, upsetting the daily routine of the youth (Aristovnik et al., 2020; de Figueiredo et al., 2021; Guo et al., 2020; Rothstein & Olympia, 2020; Sama et al., 2021) and engendering negative emotions (Brooks et al., 2020; Cao et al., 2020; Elmer et al., 2020; Ma & Miller, 2021; Zimet et al., 1988). The closure of educational institutions in order to contain the spread of the disease, has required the activation of measures to maintain continuity of learning. The Italian Ministry of Education, with the support of other organizations, has provided professional development to teachers and technological devices to students, accelerating the integration of technology in the teaching and learning process (Pellegrini & Maltinti, 2020). Due to the containment measures imposed during the state of emergency, important aspects of the lives of preadolescents and adolescents have been negatively impacted (Vicari & Pontillo, 2022), and they have had to shape their social practices (Welch, 2016) according to new times and spaces (Sabato et al., 2021). These forced changes make it even more important to gather new research evidence on adolescents’ affective experience.

In this article we take into account a peculiar concept of affective experience based on psychological theory that uses the term “affect” to refer to the underlying experience of feeling, emotion, attachment and mood (Hogg et al., 2010). The psychoanalytic origins of the concept is claimed by Andre Green (1999) who describes the affect as a complex mental phenomena in which the embodied experience is connected with an idea whereas emotions can be independent from intellectual thinking. Affects begin at the dawn of mental life, when ideas first become associated with sensations of pleasure-unpleasure. The latter are primarily connected with gratification and/or lack of gratification of drives and wishes. The development of affects and their differentiation from one another constitute an aspect of Ego development. Freud (1915) described the affect as a subjective state:

*What is an affect in the dynamic sense? It is in any case something highly composite. An affect includes in the first place particular motor innervations or discharges and secondly certain feelings; the latter are of two kinds – perceptions of the motor actions that have occurred and the direct feelings of pleasure and unpleasure which, as we say, give the affect its keynote*. (p. 396)

Within affective experience we can include feelings, emotions even as first reactions, affectional bonds and attachment that human beings live in their significant relationships since their childhood. Further current research about the concept of affect can be found in Aguillaume (2008).

Giving importance to affective experience we can ask if with distance learning has it been possible to preserve the affective ties based on face-to-face relationships, active participation, personal engagement, social interactions that characterize school life in-person.

### Schooling Experience during the Pandemic

Distance learning has had important implications both on a strictly didactic and psychological level, with a strong impact also on the relational dimension (Aguilar et al., 2020; Viner et al., 2020) bringing out in young people the thought that it is not possible to substitute the complex experience of school with only distance learning (Montanari, 2021; Pediconi et al., 2021). Adolescent, forced to restrict their relational space within the home, have suffered the effects of a collapse of boundaries in which roles and activities have merged with each other, making the distinction between the various environments, between school experiences and extracurricular experiences in general, blurred (Micalizzi, 2021; Segre et al., 2021).

Research has shown that a positive family climate, a good relationship with the teachers, and a good self-regulation of emotions were important factors in facing the difficulties due to distance learning (Pozzoli et al., 2022). Authenticity and collaboration have facilitated children’s learning, as well as supportive pedagogies and motivational strategies, improving their well-being and enabling academic progress (Yates et al., 2021). However, for the majority of children distance education increased social class academic disparities: youth with disadvantaged families (low economic resources, only one shared computer, lower quality of hardware, software and internet access, absence of individual study space, lower digital skills, the main use of digital resources for entertainment rather than for work and education, less drive for autonomy and independence) have had huge repercussions in the continuity of learning rather than those with upper/middle-class families (Goudeau et al., 2021). Studies in special education also show significant losses in the developmental and educational processes of students with a disability compared to a pre-pandemic ordinary school setting, highlighting as protective factors specific teaching tools and caring human contact, a supportive home environment, adequate Information and communications Technology (ICT) devices and a good internet connection (Capurso & Roy Boco, 2021).

However in a relevant number of cases distance learning has brought with it many psychological problems such as an increased level of restlessness, aggressiveness, anxiety, depression, irritability, boredom, inattention, a scarce commitment and autonomy during lessons in the youngest, and exacerbated the difficulty of children with pre-existing behavioral problems like autism and attention deficit hyperactivity disorder (Maggio et al, 2021; Panda et al., 2021; Scarpellini et al., 2021), in some cases it has also stimulated the resilience of young people who have chosen to occupy an active role in the choice of methods, activities and organization, using the experience of distance education to develop more profound self-regulation and call for reshaped, more collaborative roles and relationships with teachers for the future (Kovács Cerović et al., 2021). In particular, recent research has highlighted the importance of self-efficacy in developing and dealing with the experience of distance learning: adolescents most confident have emphasized their successes by making the best use of relational resources, whereas those less confident children have more strongly perceived the difficulties not only in learning but also in the more social aspects of the school (Pelikan et al., 2021).

During distance learning, students felt the need to participate in more interactive activities, inviting teachers to make every effort to transform the online “traditional” frontal teaching into more practical activities (Cadamuro et al., 2021). By meeting the teachers online every day, the students were able to maintain only very partially those informal relational aspects (physical contact and informal gestures) that characterize the pupils-teachers relationship and which are so decisive for the school experience. The voice remained almost the only one supporting online schooling relationships (Addimando et al., 2021).

### Back to School

Once the educational institutions reopened after the lockdowns, adolescents and preadolescents returned to school but did not find the same situation they had experienced before the COVID-19 pandemic, and they have adapted to the return to in-person education (Klootwijk et al., 2021). If before the pandemic the school represented an ordinary setting and gave adolescents a sense of normality and continuity, the return to post-lockdown school meant for young people a break in the “new-normal” routines that they had experienced with distance learning and families at home (Pelaez & Novak, 2020). At school they had to adapt to a new “abnormality” (Crick, 2021): made of masks, daily temperature checks, washing hands upon entry into the classrooms, frequent cleaning and ventilation spaces, break time sitting in the chair, and keeping a distance of 1.5 meters from their friends which all required psycho-emotional processing (Levinson et al., 2020).

Although generally among most students positive affections prevailed on their return to school, testimony to the success of an adaptive and dynamic process capable of coping even with sudden changes, it should not be underestimated however the difficulties, especially of those who already showed pre-existing psychological problems, special needs or disadvantages, and who returned to school with fewer resources, feeling more vulnerable and in need of more targeted support (Fegert et al., 2020; Flynn et al., 2022). Taking into account the demographic variables, it seems that the female secondary school students who experienced the return to school at a particularly critical moment reacted to the new school reality in a more pessimistic manner. (Branquinho et al., 2021; Lessard & Puhl, 2021; Pediconi et al., 2021).

Several authors have highlighted how the school could play an important role in helping children to analyze past events and identify key coping trauma strategies (Capurso et al., 2020; Dvorsky et al., 2021; Waters et al., 2021). This could be done by giving voice to adolescents and adopting a positive education following the principles of prevention-based psychology and promotion-based psychology (Slemp et al., 2017; Waters et al., 2017), so that school could become the favourite place for adolescents and preadolescents in which to process emotional critical events connected to the COVID-19 pandemic, rediscover interpersonal connections, develop an awareness of effective coping strategies, build up their resilience and minimise the risk of long-lasting difficulty. Activities that encourage classroom discussions about the event and that promote different narrative modes (e.g., story-telling, drawing, writing, fantasy) without prejudice or suggestion allow youth to express feelings and thoughts, to accept them as a normal part of their individuality and to learn new strategies through sharing and comparison with others (Capurso et al., 2020). Through narratives, youth can explore hypotheses and test different solutions (Bruner, 1986), reflect on different ways of coping with events and assess the consequences of different responses (Kratochwill et al., 2016).

Taking into account research evidence, this work aims to investigate and identify the aspects that support the school experience and distance learning, as well as the impact that their absence/presence has had on the emotional experiences of young people during the pandemic. It could therefore provide important information on how to best invest efforts and resources in future emergency situations.

## The Present Study

Recent studies about “school hesitancy” (Bushweller & Lloyd, 2021; Goldstein, 2021; Polikoff et al., 2022) point out the resistance of families to leave the established routines based on remote learning. Some are reluctant to interrupt their newfound stable alternatives for a return to school shaped by hybrid schedules — with students in class some days of the week and working from home on others — in facing the risk of closures and quarantines.

We can observe that some teenagers, even knowing their schools had reopened, started to feel that remote learning works better for them. As a consequence, superintendents and school leaders are now in a bind. Most believe that endless remote school poses health and social risks for kids, but they do not want to pressure families. Continuing adherence to social-distancing guidance also means most schools cannot operate at full capacity, and staffing remains an issue.

Most researchers asked teachers and families about the well-being, motivations and health of adolescents during distance schooling in the pandemic period (Scarpellini et al., 2021; Toseeb et al., 2020). Although the transition from pre-pandemic school to post-pandemic school implied an entire new educational system, only limited research has been conducted in which adolescents have been asked directly about their school experiences (e.g. Duckworth et al., 2021; Fisher et al., 2021; Holzer et al., 2021; Klootwijk et al., 2021; Montanari, 2021). Indeed, at the current time research in literature lacks data about how the aspects of school routine, relations, and moments experienced before the pandemic have favoured or hindered the return in person, and the experiences related to distance learning. The present study aims to address this gap by answering three research questions about the school experience described by students themselves:

- RQ1 – What aspects of schooling experience before the pandemic did students hold when they returned to their desks after distance learning? Both the formal and informal aspects of the school routine will be analyzed.
- RQ2 – What was the students’ schooling experience during distance learning? Middle and high-school students will be considered.
- RQ3 – What components of students’ schooling experience promoted or impeded their return to school in person? It will be analyzed how distance learning influenced the affective experience linked to the return to in person classes.

## Material and Methods

### Sample and procedure

The research involved 157 students (50, 3% male and 49,7 % female) with a mean age of 13,58 years old (SD = 2,107) from central Italy. In order to compare the experiences of preadolescents and those of adolescents, participants were selected from lower secondary school (Age 11–13, N = 87, 48 females and 39 males) and upper secondary school (Age 14–18, N = 70, 30 female and 40 males). The sample didn’t include students with the declared conditions of fragility (i.e. hospitalized students, students with mental health issues, learning difficulties, language difficulties, and foreign students).

The data were collected in-person both in lower and upper secondary schools (October 2021) after the reopening of schools ended the acute phase of the health emergency from COVID-19. The anonymous questionnaire was dispensed during school hours by two school psychologists after obtaining informed consent from parents and assent by the participants. The activity was approved by the local school board.

### Measures

In line with the recent studies about online distance learning (Kovács Cerović et al., 2021; Mishra et al., 2021) and on the impact of the COVID-19 pandemic on the experiences of young people (Branquinho et al., 2021; Scott et al., 2021; Yates et al., 2021), we prepared an ad hoc questionnaire inspired by the Sacks and coll. “Sentence Completion Test” (Sacks & Levy, 1950). It was administered in October 2021 in person, when the state of emergency was lifted and schools were reopened. It consisted of two types of items: “sentences to complete” relating to the teenager’s experience of school during the quarantine (distance learning) and after strict emergency state (i.e. “The return to school in September 2020…”, “When I think of DAD/DID…”, “My teachers…”) and multiple-choice questions for demographic and educational variables. In particular, the questionnaire was used to investigate:

- daily school routine including both informal (recess and relationships with classmates) and institutional school moments (oral testing and relationship pupils-teachers) to answer RQ1;
- emotional experience of distance learning to answer both RQ2 and RQ3;
- emotional experience of returning to school to answer RQ3;
- who adolescents and preadolescents spent time and space with during the lockdown to answer RQ3.

Demographic and educational variables were the type of school, age, and gender we con. All responses were classified as “positive”, “neutral” or “negative” by two independent observers-raters, both psychologists with professional experience in the research field, with an inter-rater reliability of 0.81. Any disagreements regarding attribution were discussed until a consensus was achieved. Specifically, the responses were classified as positive when the completed sentence showed positive experiences or feelings towards the subject of the proposition (i.e. “When I think of DAD/DID I think that I had less anxiety and it was easier for me to follow the lesson”), as negative when the completed sentence showed negative experiences or feelings towards the subject of the proposition (i.e. The return to school in September 2020 was stressful and awful for me”). Sentences that did not have any affective connotation were classified as neutral (i.e. “My teachers were mostly women/as always).

To deepen the analysis of the affective experience that supports the school one, a general synthetic indicator has been constructed by adding the evaluations that adolescents and preadolescents have reserved for the individual components of the daily school routine and it has been labelled the “feeling of the school”. In particular: positive responses have been encoded as +1; negative responses have been coded as -1; neutral responses have been coded as 0. For each participant, a numerical indicator was thus obtained, represented by the algebraic sum of all his/her evaluations, called “feeling of the school”.

### Data Analysis

Psychometric analyses were conducted through the IBM SPSS Statistics statistical software version 25.0. Data analysis was performed using frequency distributions for categorical variables, Anova, T-test and Pearson correlations for numeric variables.

To answer RQ1 the a Chi-Square test was performed on differences between adolescents and preadolescents about feelings towards the components of daily school routine: oral testing, recess, and relationships with classmates and teachers.

To answer RQ2 Pearson correlation and test of Chi-Square were performed to detect differences between adolescents and preadolescents about feelings towards distance learning; in addition, a test of Chi-Square was performed to analyse feelings about distance learning matched by the affective relationship with teachers and by the informal routine during the recess.

To answer RQ3 a test of Chi-Square was performed to detect the feelings linked to returning to school matched by age (adolescents and preadolescents), the experience of distance learning, the relationship with classmates and by other aspects of informal routine (recess). Chi-Square was also performed to analyse the influence of attachment figures with which the children experienced the quarantine on the feelings related to going back to school. A T-test was performed to detect a significant difference in averages of “general feeling of school” related to experienced distance learning. Anova was performed to analyse how the general feeling of school changed based on age (adolescents and preadolescents).

## Main Results

### Daily School Routine in Continuity pre-post Pandemic Period

In this first section the findings presented are related to RQ1 about what aspects of schooling experience before the pandemic the students had held when they returned to their desks after distance learning schooling. Both the formal and informal aspects of school routine will be analysed.

**Figure 1:**
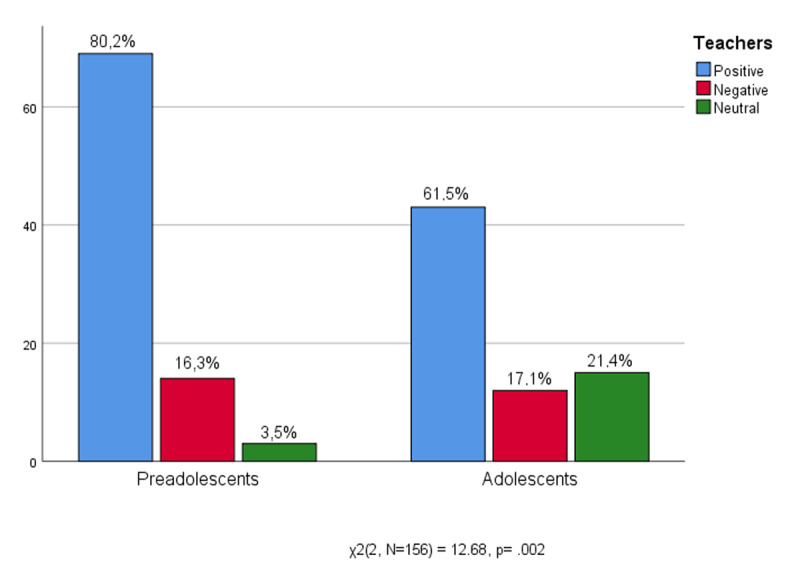
Age * Teachers.

As can be seen in Figure 1, once back at their school desks the middle school students (preadolescents) consider the relationship with the teachers very important (80.2% rate them positively) and seem less willing to note any shortcomings or defects, indeed only 16.3% express a negative opinion. For pre-adolescents an affectionate bond with teachers is confirmed as an extension of emotional ties with significant figures even at school. High school students estimate their teachers (61.5% positive) as well, although a not insignificant number prefers to maintain a position of neutrality (21.4%) that could conceal more critical comments. It should not be forgotten that for high school teenagers the relationship with teachers becomes challenging as an expression of a growing confrontation with adult figures (Cooper, 2002; Wentzel, 2009).

**Figure 2:**
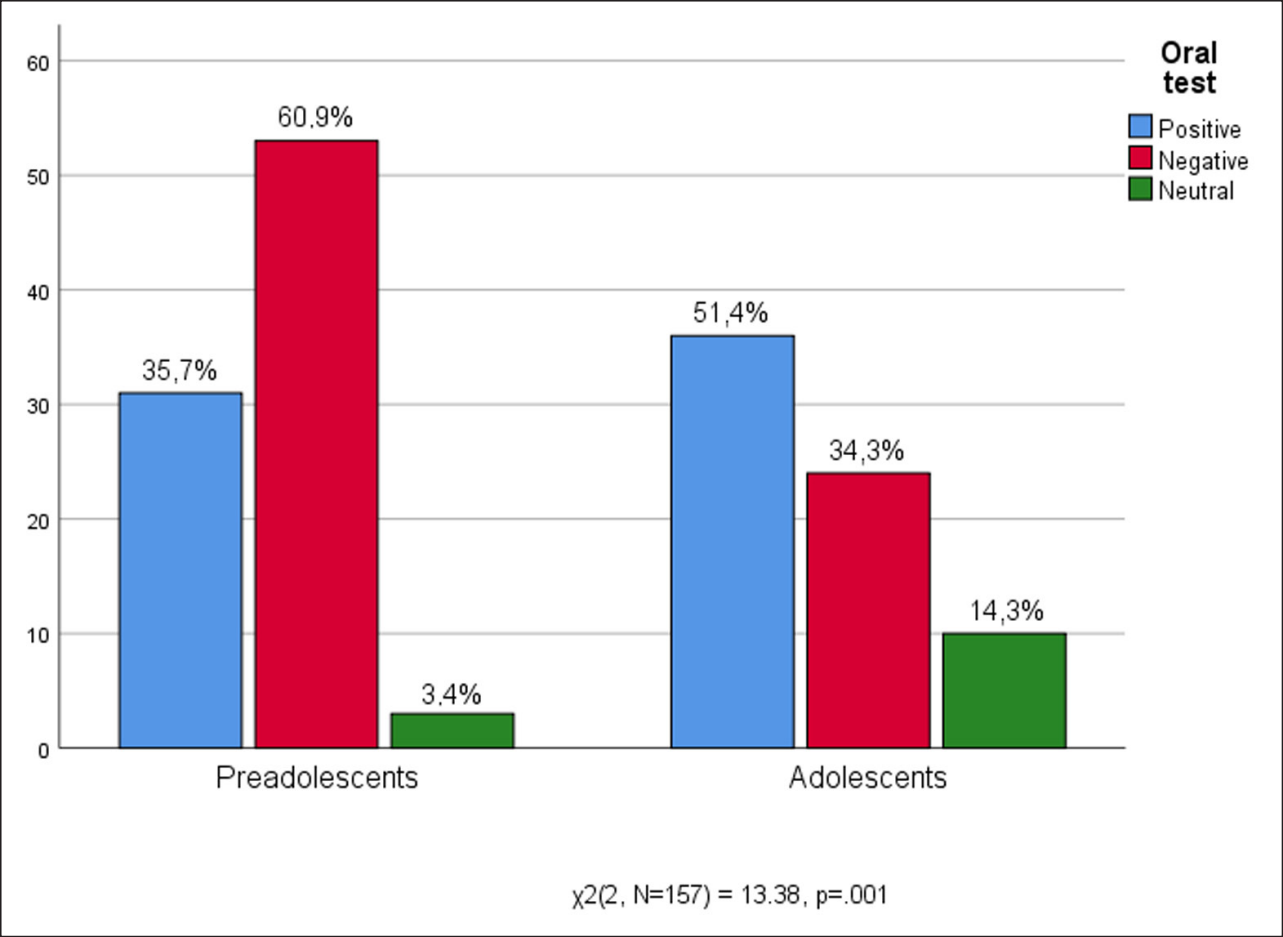
Age * Oral test.

Considering the institutional aspects of school experiences, Figure 2 shows that the experience of oral tests improves with age. In an oral test the teacher asks several oral questions to the student in order to evaluate his/her preparation on specific topics. Preadolescents are in greater difficulty than high school boys (60.9% vs 34.3%). Most middle school students have a negative and anxious attitude towards oral tests, which they see as challenging not only their academic performance but also their self-esteem. On the contrary, adolescents, while not hiding experiences of difficulties during the oral test, seem ready to take up the challenge, and are more willing to test themselves (51.4% vs 35.7), in fact there are just a few who neutralize the feelings related to being evaluated (14.3% vs 3.4%).

**Figure 3:**
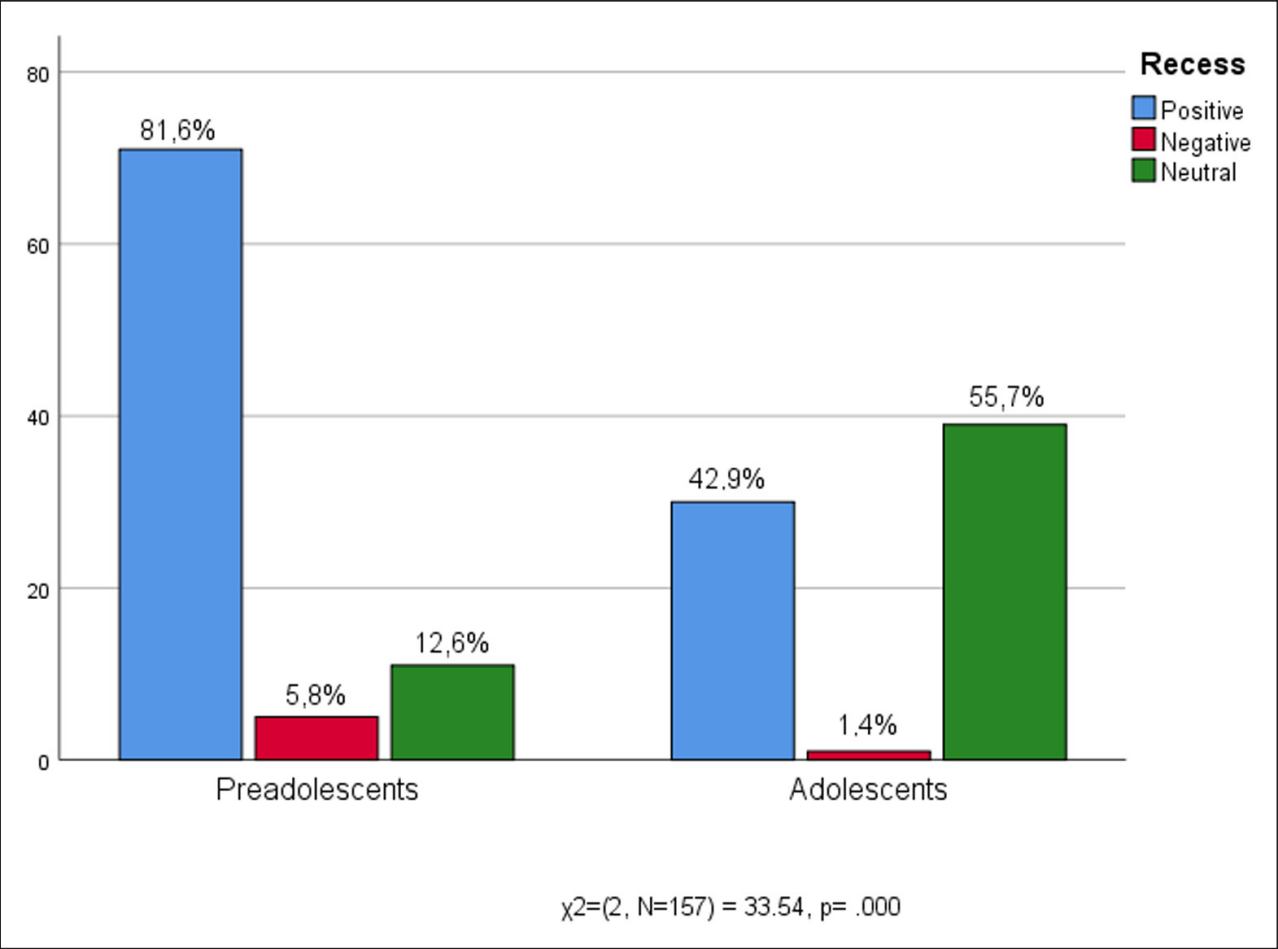
Age * Recess.

Among the informal aspects of the school routine, for sure it is recess that has a privileged position – it is the pre-adolescents who are most enthusiastic about it, even if there are good experiences also among the adolescents. Figure 3 shows that very few speak badly of recess, yet among adolescents there is a greater impatience, as shown by the high number of neutralizations (55.7%) concerning break times. Adolescents that neutralized judgement could therefore hide a certain emotional distance.

### What Affected the Distance Schooling Experience

This second section presents the findings related to the second research question (RQ2) about what was the students’ experience, both for preadolescents and adolescents, of distant learning during the pandemic. Students’ approval rating of distance learning matched by age will be statistically analysed.

**Figure 4:**
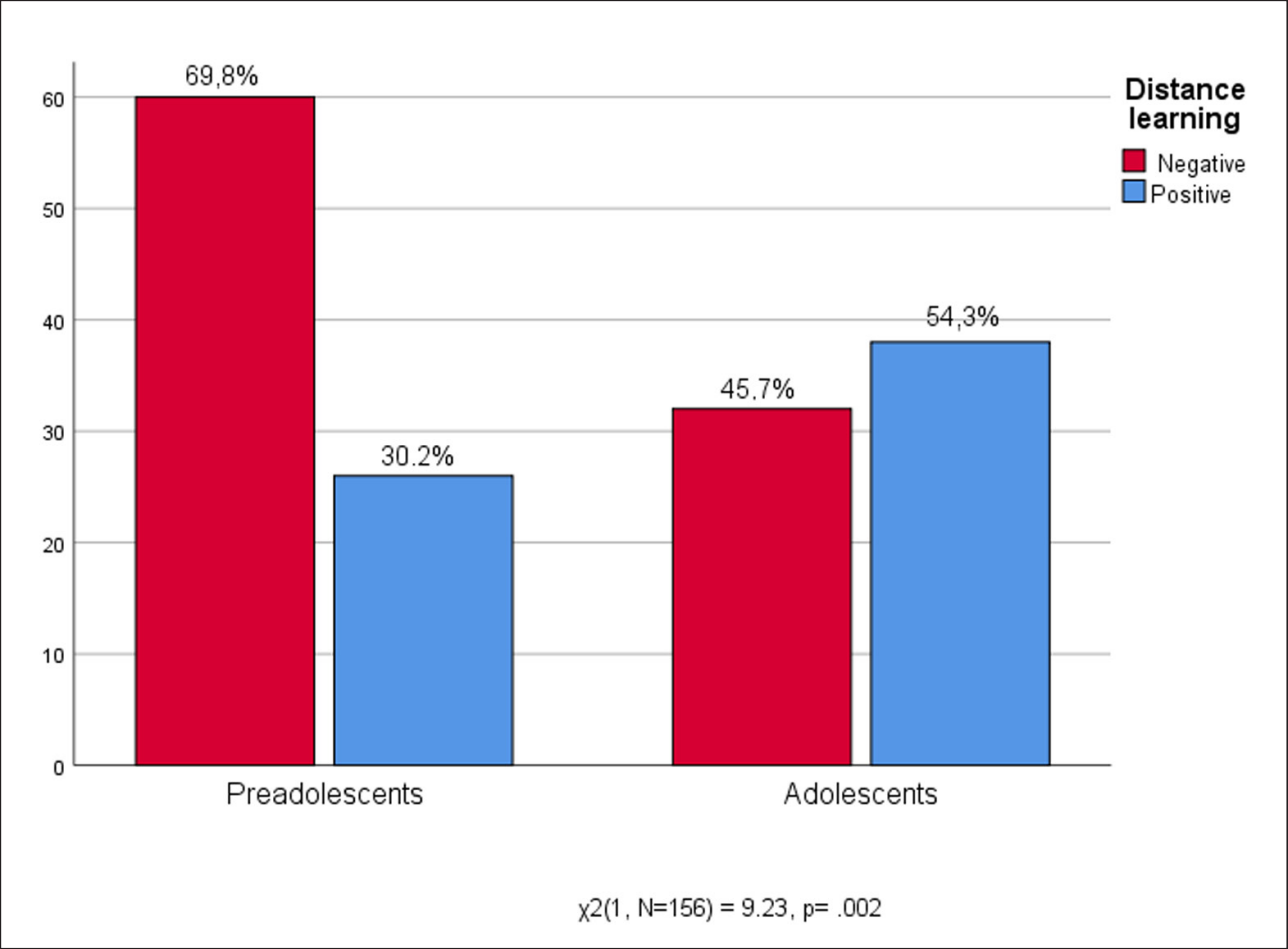
Age * Experience of Distance Learning.

The statistical analysis of the correlation between the experience related to distance learning and age (r(154) = .27, p = .001) shows that the positive evaluation of distance teaching grows proportionally to age. The high school students appreciated it more. The significant differences in Figure 4 also confirms this fact: the vast majority of middle school students (69.8%) have negatively assessed distance learning, highlighting problems and disadvantages, while among the students of high school there are slightly more of those who rate it positively (54.3%). Both preteens and adolescents had to process their surprise about the school interruption due to the pandemic, only partially replaced by distance learning.

In the literature there are interesting qualitative gender differences relating to the lockdown experience (Capurso et al., 2023; Capurso et al., 2022): data report that females missed school, family and relatives, hanging out, and having direct contact with others more than males. Adolescent girls present higher emotional sensitivity to stressful life events, and consequently, they tend to report higher levels of attachment to peers and favour quality intimate relationships. Instead, especially among middle school students, males missed sport and hobbies but were helped by video games and TV. Other studies found that females experienced more negative emotions during the distance learning experience (Cadamuro et al., 2021). Despite many researches showing important results about gender differences, our statistical analysis did not notice them.

**Figure 5:**
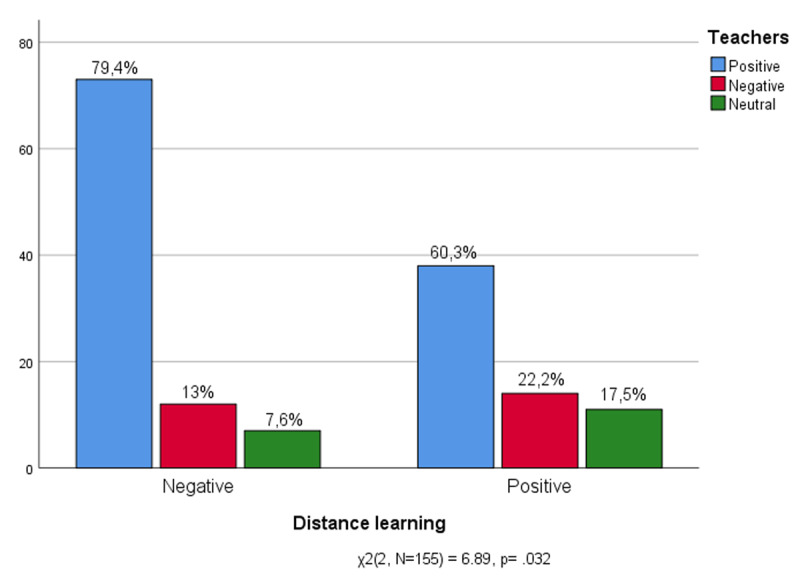
Experience of Distance learning * Teachers.

Figure 5 shows the links between the evaluation of students’ relationships with teachers (positive, negative, neutral) and their experience of distance learning (positive, negative). Among those who have negatively experienced distance learning as a new way of doing school, there are many who express a positive opinion about their teachers (79.4%). Those who have enjoyed distance learning (60.3%) also have a good relationship with teachers, but the number of those who feel negative towards them (22.2%) or prefer to remain neutral (17.5%) is increasing.

**Figure 6:**
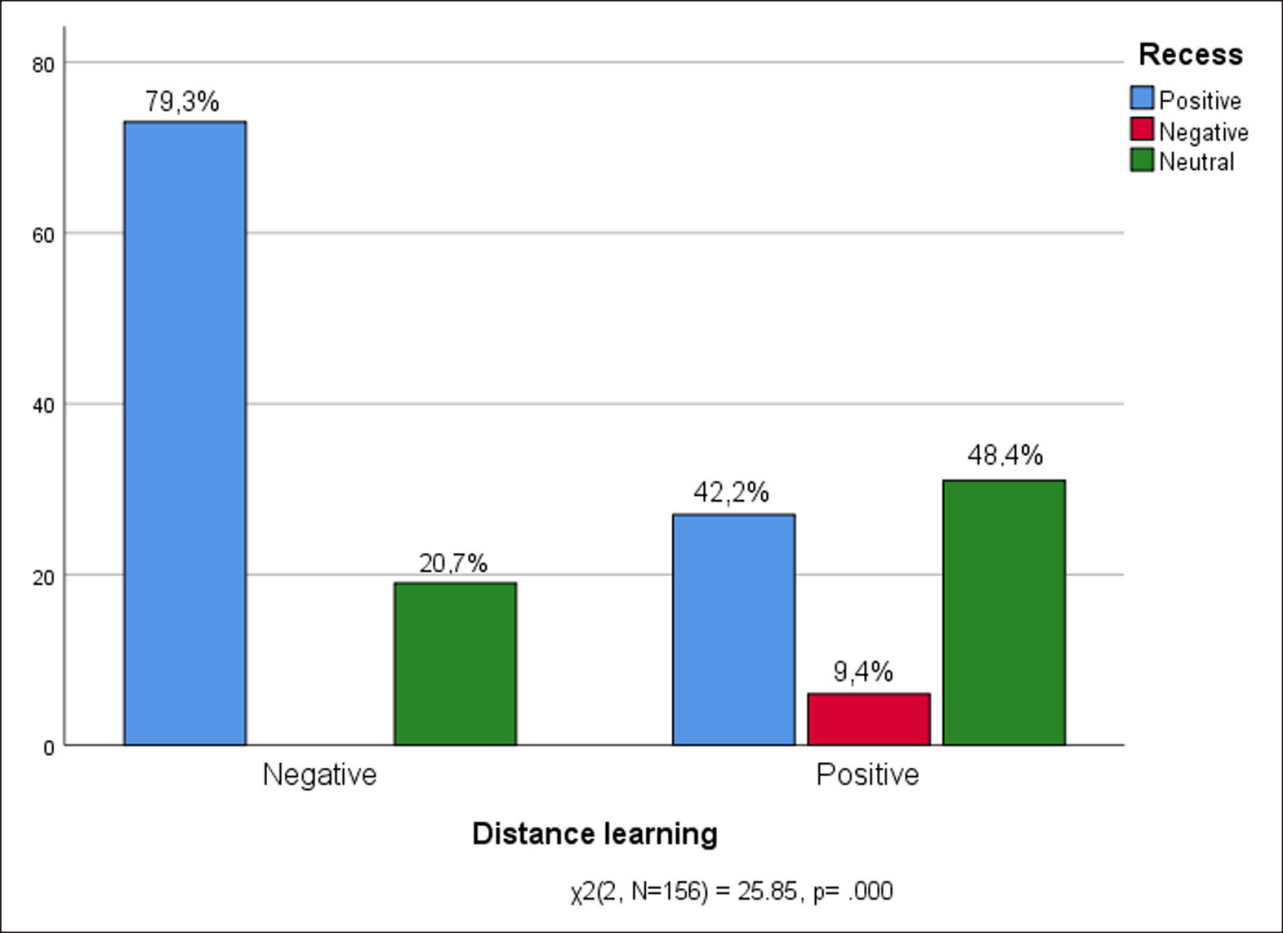
Experience of Distance learning * Recess.

As we have seen, the moment of recess remains a cornerstone of the traditional school experience, but it is interesting to note how feelings related to recess are influenced by feelings related to the experience of distance learning. In Figure 6 it can be seen that most of those who have struggled with distance learning appreciate moments of recess (79,3%), when the school experience moves into the corridors. Only a small minority prefer not to express themselves (20.7%), while there is no negative opinion on recess among those who have negatively experienced distance learning, confirming the fact that recess is a milestone of the school experience, but in person. On the contrary, most of the young students who rated distance teaching positively preferred to avoid expressing an opinion about the moment of recess (48.4%), a good number expressed a positive response (42.2%). Note the few who do not appreciate recess at all (9,4%). Distance learning has taken away from children a distinctly social and recreational aspect of the educational experience.

### Emotional Experience of Returning to School

**Figure 7:**
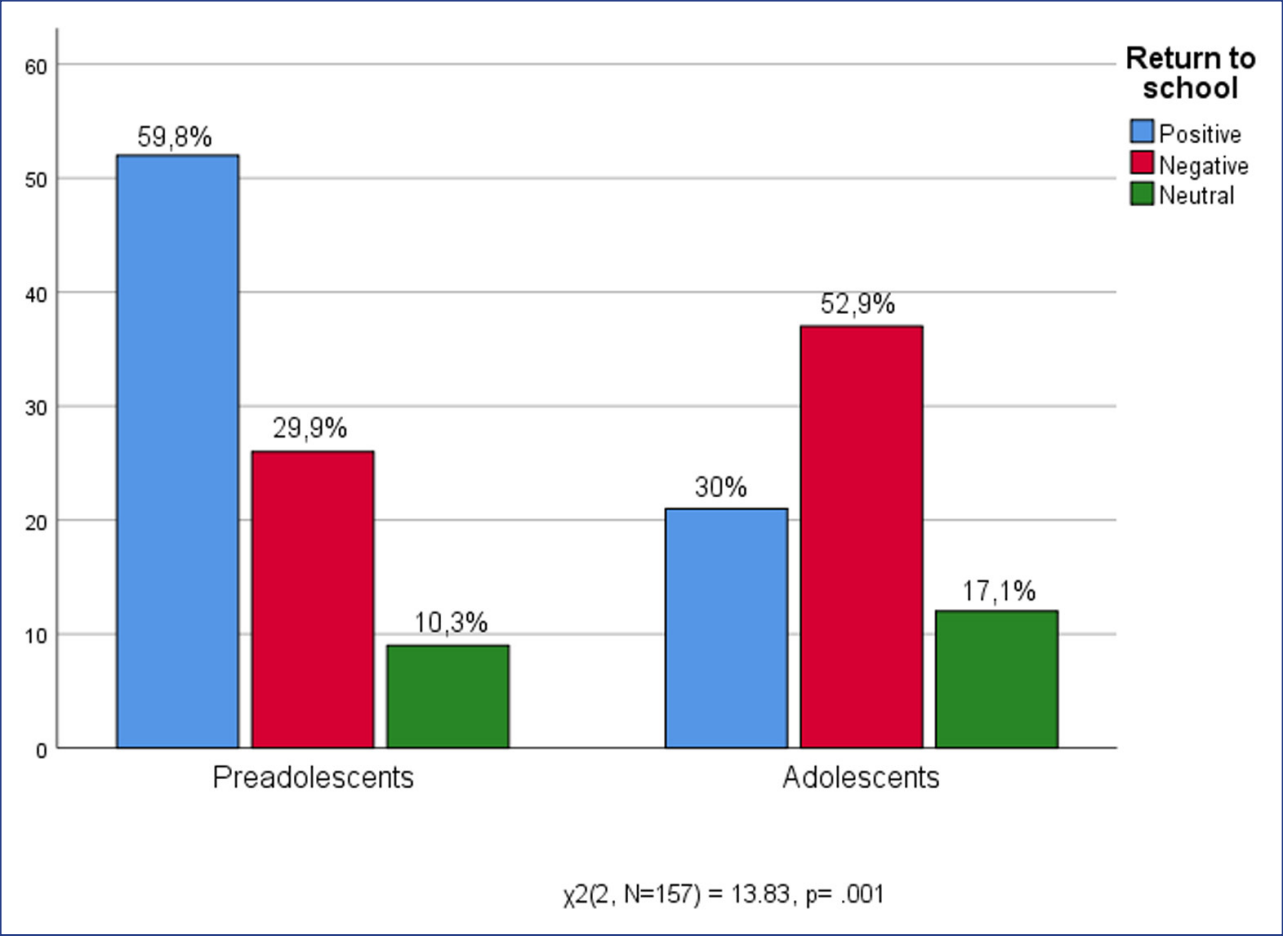
Age * Return to school.

In this section we present findings related to the third research question (RQ3): what components of schooling experience promoted or impeded the return to school and how distance learning influenced the affective experience of returning to school. The results highlight significant differences between adolescents and preadolescents (Figure 7) showing how students experienced their return to school. Most preadolescents of the sample (59,8%) experienced the return to school as a positive moment. Preadolescents were enthusiastic about going back to school, reaffirming the centrality of the school experience in their social life.

Most adolescents of the sample (52,9%) negatively described going back to school. Older students seem to have been more affected by the interruption caused by the pandemic. The results show significant different attitudes about returning to school between preadolescents and adolescents. Preadolescents experience a profound extension of social and emotional relationships thanks to school. However, for adolescents school instead challenges their emotional experience and success.

**Figure 8:**
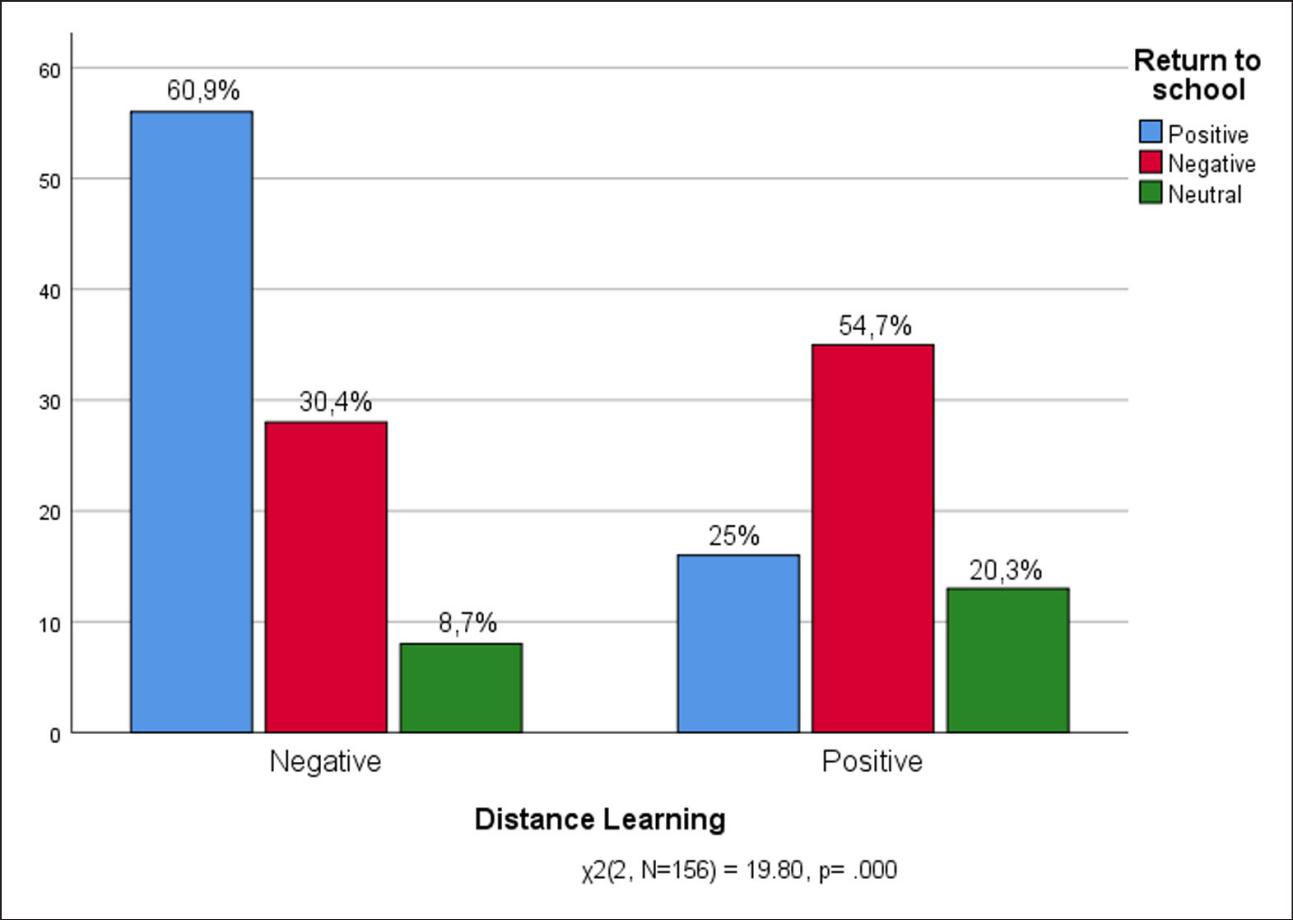
Experience of Distance learning * Return to school.

Now, one might wonder whether the experience of distance learning has encouraged or hindered the return to school in person (Figure 8). The figure shows that most of the students who have experienced distance learning as a negative interruption express a very positive response on returning to school in person (60.9%). On the contrary, the majority of students who have enjoyed distance learning have struggled to return to school in person (54.7%): for them distance learning has hindered their return to normality. For most of the sample the distance learning and the learning in person are contrasting experiences. Those who favour one despise the other and vice versa. What about the children who have experienced distance learning badly and now are not even happy to return to school (30.4%)? They are the students who have experienced the interruption in a traumatic way – the pandemic interrupted the time of school and therefore the school of the past no longer exists.

It could be read in the light of considerable uncertainty also the number of those who effectively neutralise their return to school, and refrain from judging it positively or negatively. In addition, they are more numerous precisely among those who say they have experienced distance teaching positively (20,2%). It could be said that the students tried to replace the in-person mode with distance learning, they tried to pay some attention to school even during the quarantine, but the online mode has not kept its promise and today these students find it difficult to return to normality, precisely those who have invested more in digital novelty.

The data indicate that it is not the institutional components of the school experience (oral testing and relationship pupils-teachers) that hinder the return to school. Indeed, in matching feelings of returning to school and the perception of oral tests, were not found any significant results. These students are not afraid of questions and the more rigorous aspects of the school experience do not prevent them from seeing the school first of all as a living environment. Rather it is among the informal components of the daily school routine (recess and relationships with classmates) that can be found the inconvenience that weighs down feelings of returning to school.

**Figure 9:**
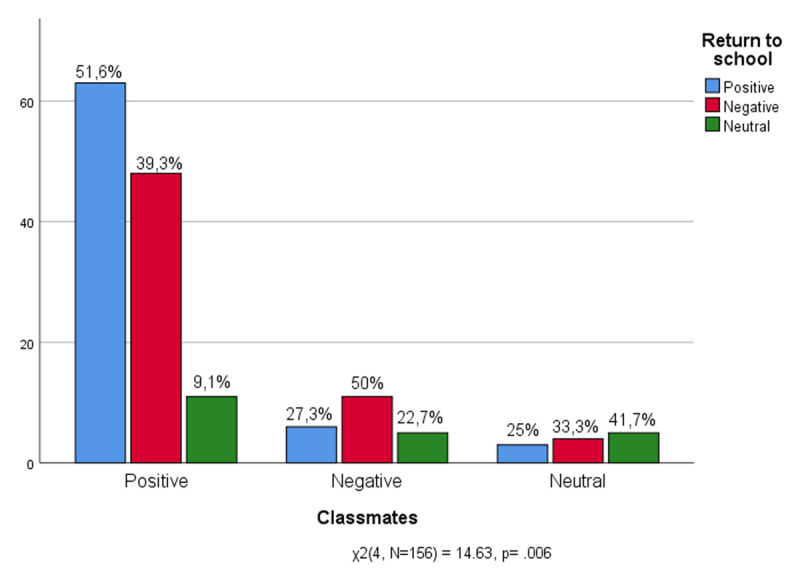
Classmates * Return to school.

Data (Figure 9) confirm that for adolescents the school is the place of social life. Being able to go back to meet friends and share life with them is the main desire of those who have experienced a positive return to school (51.6%). Although among those who return to school willingly, are many who grow dissatisfied with classmates (39.3%). The difficulties among peers and with classmates are shared, as could be expected, even by those who have negatively experienced the return to school (50%).

**Figure 10:**
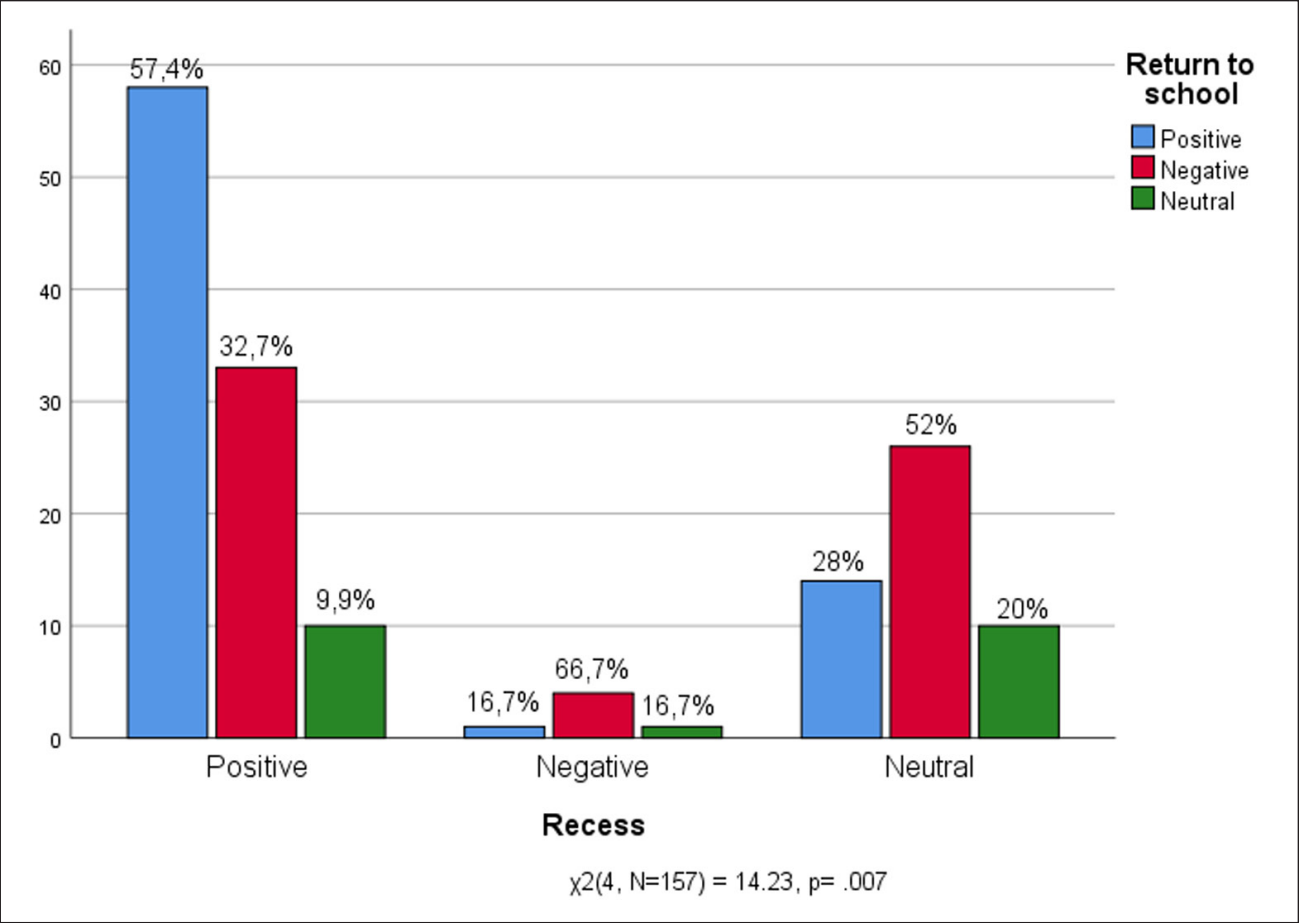
Recess * Return to school.

Keeping in mind the informal components of school experience, in Figure 10 it is observed the relationship between the moment of recess and the experience of returning to school. Those who returned to school willingly could not wait to return to the enthusiasm of the bell that signals the break between lessons (57.4%). There are some who do not appreciate recess (32.7%) and only a minority who prefer not to respond (9.9%).

The figure for those who have not returned willingly to school is fairly similar: most do not appreciate the moment of recess (66.7%). The moment of recess is not appreciated even by those who maintain a neutral position towards returning to school (52%).

xAnalysing the emotional complexity of the return to school, it could be important to note how the relational figures that accompanied the lockdown supported the return to normality.

**Figure 11:**
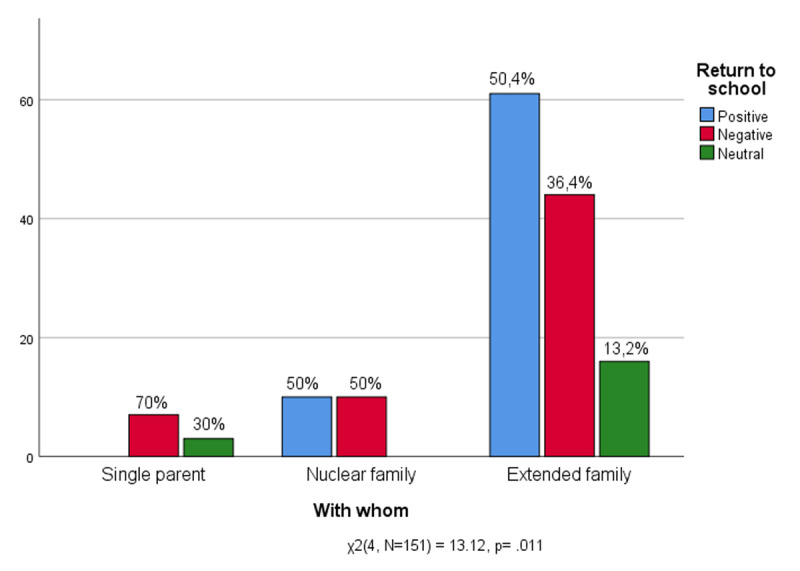
With whom they spent the lockdown * Experience of Return to school.

Figure 11 shows the relationship between the attachment figures with which the children experienced the quarantine and the feelings related to going back to school. Among those who have experienced quarantine with only one parent, none express a positive opinion on returning to school. This data could indicate the greater relational difficulty of the children who have experienced the quarantine in close contact with a single figure of attachment in returning to the social life that the school represents. For these teens, the return to school seems to have represented the breaking of a symbiosis which, however, denounces regressive impulses that risk slowing down the conquest of emotional independence. A fact that could be read as a resistance of these teens to come out of a kind of regressive affective shell, a tête-à-tête that confinement may have fuelled. Those who have spent lockdown with both parents and any siblings (nuclear family) are equally divided into positive and negative feelings about returning to school between difficulty and desire. They interpret on the one hand thoughts of difficulties about returning to normality, and on the other, the desire to return to social life. It is the extended family (composed of parents, children and other relatives or cohabitants) that supports a clear majority of children who have positively experienced the return to school (50.4%) even if they are accompanied by a certain percentage that has had a negative experience (36.4%). Only a minority prefer to abstain from evaluation (13.2%). For students of the sample the sharing of lockdown with more than one attachment figure seems to have facilitated the return to social life.

To deepen the relevance of the affective dimension that supports the school experience, a synthetic affective indicator has been developed, resulting as the algebraic sum of the evaluations of the individual components of daily school routine (both informal and institutional school moments), which connotes the general feeling with which each student invests the school experience. The findings show that the experiences related to distance learning are significantly related to the general feeling of the school. Indeed, T test (t(153) = –4.393, p =.000) shows a significant difference in averages of “general feeling of school” between those who have positively and negatively experienced distance learning: they are those who love school (M = 4.00, SD = 2.95) who suffered a lot from the transition to distance schooling. By contrast, the children who are less keen on school (M = 1.87, SD = 2.97) tolerated distance teaching better.

**Figure 12:**
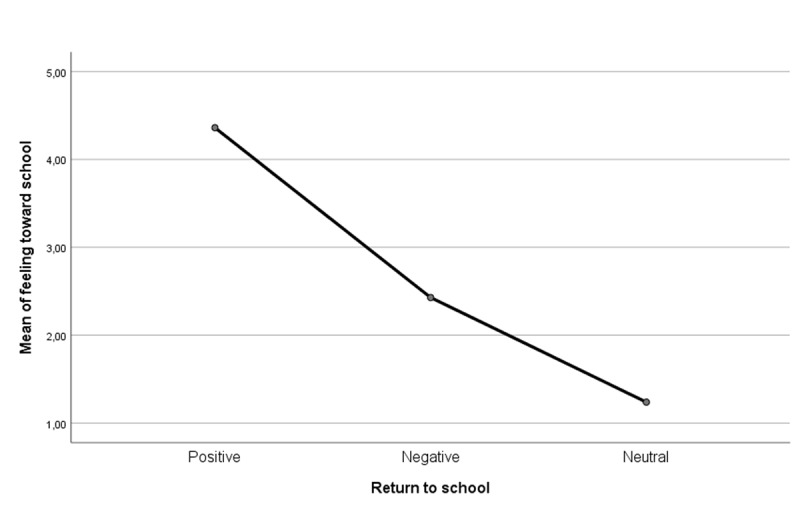
ANOVA – Return to school * Feeling toward school.

The feeling for school becomes an important indicator also to interpret the experience of the return to school of adolescents and preadolescents. In Figure 12 it is possible to observe the chart of the averages (F(2, 153) = [12,565], p = .000) relative to the match between feeling for school and going back to school.

Those who reserve special affection towards the school (M = 4.36, SD = 2.67) are excited to go back. Less enthusiastic are the students who are less passionate about school (M = 2.42, SD = 3.16). The data relating to students who have a low feeling towards school (M = 1.23, SD = 3.01) is more complex, and who abstain from evaluating their return to school in person. An abstention that could be interpreted as an implicit confirmation of the fact that the neutralized feelings conceal difficulties of an affective nature. Tukey’s HSD Test for multiple comparisons found that the mean value of feeling for school was significantly different between students who had positive feelings toward distance learning and those who lived it with more sorrow (p = .001, 95% C.I. = [0.7379, 3,1272]). Also, a significant difference was detected between students that appreciated distance learning and those that neutralised feelings about it (p = .000, 95% C.I. = [1.4056, 4,8405]). Instead, there was no statistically significant difference in mean scores of feeling for school between those who experienced distance learning as negative moments and those who reported neutral feelings (p = 0.243). Those who have experienced an emotionally flattened or more markedly negative return would therefore seem to have something in common: an affect for school impoverished by the pandemic or experienced as potentially painful that hinders them in returning to invest positively in school.

## Discussion

### RQ1: Daily School Routine in Continuity Pre-post Pandemic Period

In view of the fact that the children spoke about school experience in general at a time when the precautionary rules dictated by the pandemic were still very restrictive, upper school students report a certain intolerance to adapt to the new “abnormality” (Crick, 2021), made up of wearing masks, washing hands upon entry into classrooms, sitting chairs at recess, as well as maintaining a 1.5-meter distance from their friends (Levinson et al., 2020). The adolescents of our sample that did not explicitly express their perplexities could experience a certain annoyance about the new forms of recess in which, due to the post-pandemic, socialisation must be forcibly “contained”. Their neutralised judgement could therefore hide a certain emotional distance.

In particular, our results show that the continuity of the pre-post-pandemic school experience has been supported by both formal aspects such as oral tests and informal school routines such as recess. On the one hand, teachers have taken on a leading role confirming themselves as the main focus of the school experience both during distance learning and as guarantors of continuity in the moment of come back to school (Guo et al., 2020; King, 2017; Marini et al., 2023), as was school in person although it has been strongly influenced by Covid safety measures. On the other hand, affectional bonds with teachers and memories of the schooling routine both formal and informal had given continuity to the school experience even during the pandemic, underlying the importance of school as the most important place for the socialisation of pre-teens and adolescents.

### RQ2: What Affected the Distance Schooling Experience

Both preteens and teenagers had to process their surprise at the interruption of school activities due to the pandemic, which were only partially replaced by distance learning/teaching. Distance schooling was experienced more resiliently by high-school students. Only a few have experienced the interruption of the school in a traumatic way: they did not appreciate either distance learning, or the return to school, reporting a kind of blackout of the school experience altogether. Other students have tried to replace the in-person mode with distance learning, paying some attention to school even during the quarantine. However, for them the online mode has not kept its promise. Those who have invested more in digital innovation, found it difficult to return to normality. For all, socialisation mediated by school experience was decisive as a support to the resumption of ordinary life after the pandemic.

Teachers remain the pivot of school experience even at a distance, assuming that positive feelings towards them have mitigated the effects of negative experiences related to distance learning (Marini et al., 2023). On the one hand, the results are consistent with other research (Cadamuro et al., 2021; Kovács Cerović et al., 2021) about the more collaborative roles and relationships with teachers. During distance learning, students participated in more interactive activities, inviting teachers in order to transform the frontal instruction into something more involving. On the other hand, those who had more detached relationships with teachers, probably due to the impossibility of having a relationship with teachers in person in which the voice remained the only one support of online schooling relationships (Addimando et al., 2021), may have had negative feelings tied to online learning.

Distance learning has taken away from the youth a distinctly social aspect of the schooling experience. Students struggling with distance learning missed informal moments of schooling in person (recess) while students better adapted to new modality at distance tended to neutralise feelings related to social aspects of school. This suspension could be read on the one hand as an effect of uncertainty that distance learning has thrown on the school in general. On the other hand, it could be read as emotional distancing from the memory of an aspect of the school routine left in the past.

Recess is undoubtedly the moment of school when students play and physically meet with peers; with distance learning teenagers have lost all that interpersonal dynamic dimension – friendships and peers, freedom, autonomy, hanging out, going out to play, physical contact – that transform school life in the arena where students undertake sense-making of the events and actively take part in a social and developmental network (Capurso et al., 2023).

A warning for the future: the maintenance of learning modalities and the spread of impersonal teaching could heavily compromise the feeling towards school of the young generation, endangering the most important experience for education and socialisation of teenagers and preteens.

### RQ3: Emotional Experience of Returning to School

In line with other studies, our results showed that preadolescents and adolescents experienced coming back to school as a delicate moment, and the new school reality in a more pessimistic way (Branquinho et al., 2021; Lessard & Puhl, 2021; Pediconi et al., 2021). The results showed significantly different attitudes about returning to school between preadolescents and adolescents. On the one hand, preadolescents willingly come back to school showing profound openness and extension of social and emotional relationships. On the other hand, most adolescents of the sample negatively described going back to school, they seem to say: “School can never be the same again!”. The previous experiences of adolescents relating to school in person could come into conflict and comparison with the new “abnormality” (Crick, 2021). Older students seem to have been more affected by the interruption caused by the pandemic, the new school has tested their emotional experience and success. They considered it a broken time that could have negatively compromised their school feeling and their feeling of self, going back to school means going back to facing new questions, confronting adults and professors who expect them to learn new information, and peers who expect new interactions (Scott et al., 2021). This data confirms the impossibility of replacing the complex experience of school only by means of distance learning (Lara-Prieto et al., 2021). It could provide further evidence of the long-term effects of the pandemic, during which pre-adolescents and adolescents were forced to restrict their relational space with deleterious effects on the distinction between the various living environments, a consequent indistinct spread of school dropout, and a certain disaffection in daily commitments (Micalizzi, 2021; Montanari, 2021; Scarpellini et al., 2021).

What favours the desire to go back to school and what slows it down? Do the students want to go back to meeting their classmates in person or are they afraid to return to the routine that forced them to get up at dawn and cope with going on school trips and the slowness of everyday real life?

The return to school becomes more exciting for those who have experienced distance teaching in a negative way, while those who felt comfortable in front of the screen experienced being frightened, shocked or disturbed returning to school. The pandemic experience seems to have complicated the indispensable relational commitments during pre-teens and adolescence (Vicari & Pontillo, 2022), a time when relationships with classmates are a blessing and a curse of the school experience (Foulkes & Blakemore, 2021), especially when they meet each other again at school.

Most of those who declared neutral or negative feelings thinking about the school moment of recess have not returned willingly to school. This could signal a despondency of those teenagers who seem to almost protect themselves from moments of school socialisation, showing some difficulty in the resumption of ordinary life after the pandemic.

The composition of the family with which the student shared the quarantine was another aspect that affected the return to school. The extended family has given greater social and interpersonal continuity for adolescents, confirming the family as a factor of resilience even in the COVID-19 emergency (Cimino et al., 2021; Maftei et al., 2022). However, it cannot be excluded that the positive feelings for the return to school for children who have been quarantined with a large family has been favoured by a new-found freedom, even more appreciated after suffering the stringent and forced sharing of time and space.

Also the analysis related to the general feeling toward school gives important information about the return to post-pandemic school. Those who experienced the positive return seem to have clung to the feeling for school keeping it alive within them, while those who returned to school without enthusiasm (negative or neutral return to school) they may have been disappointed by too high expectations that were not met by the restrictive measures. Disappointed students risk developing a defensive feeling (negative or neutralized) that detaches them from a school experience (Altwaijri et al., 2022; Leuzinger-Bohleber & Montigny, 2021).

## Conclusion

In conclusion, the results show that even though online learning has been a useful tool for delivering didactic (theoretical) concepts, it has been unable to replace conventional schooling and education. However, despite the students and teachers being overwhelmed at the beginning of the pandemic, they have shown remarkably resilient aspects of schooling during different phases of contagion.

About distance learning the students tried to replace the in-person mode with distance learning. However, the online mode has not kept its promise: in particular those who have invested more in digital novelty seem to find it more difficult to return to normality.

From the perspective of affectional bonds, if the family, teachers, and memories of social moments of school routine are confirmed as the protective factors during the quarantine that have favoured the return to life in person, distance learning seems to have weighed down the children’s feeling towards school experience as such. This result prompts us to reflect on the future, that is the maintenance of impersonal learning methods could seriously compromise the emotional investment of students in school, penalising the most important educational and social experience for the younger generations.

## Limits of the Study

Despite clear contributions, some limitations to this study should be noted. The small size of the sample and the non-randomized recruitment do not allow the results to be generalized to the whole population. A bigger sample would allow for more sophisticated statistical analysis. Furthermore, these findings are inevitably limited to the chosen variables.

## Implications for Further Research

Other variables related, for instance, to students’ individual charateristics, conditions and dispositions might be relevant in explaining the found associations in this paper and thus should be considered in future investigations. It would also be interesting to expand the research to populations at risk or disadvantaged (hospitalized students, students with mental health problems, learning difficulties, language difficulties, foreign students) to be able to compare the results with the emotional experiences of pre and post-pandemic school for youth in conditions of fragility.
